# Short-term effects of moderate intensity physical activity in patients with metabolic syndrome

**DOI:** 10.1590/S1679-45082013000300011

**Published:** 2013

**Authors:** Caroline Macoris Colombo, Rafael Michel de Macedo, Miguel Morita Fernandes-Silva, Alexandra Moro Caporal, Andréa Emilia Stinghen, Costantino Roberto Costantini, Cristina Pellegrino Baena, Luiz Cesar Guarita-Souza, José Rocha Faria-Neto

**Affiliations:** 1 Pontifícia Universidade Católica do Paraná, Curitiba, PR, Brazil; 2 Hospital Cardiológico Costantini, Curitiba, PR, Brazil; 3 Universidade Federal do Paraná, Curitiba, PR, Brazil

**Keywords:** Metabolic syndrome X, Exercise, Risk factors, Obesity, Inflammation

## Abstract

**Objectives:**

To evaluate whether a short-term moderate intensity exercise program could change inflammatory parameters, and improve different components of metabolic syndrome in sedentary patients.

**Methods:**

Sixteen patients completed the 12-week program of supervised exercise, which consisted of a 40 to 50 minutes of walking, 3 times a week, reaching 50 to 60% of the heart rate reserve. The parameters evaluated before and after intervention were waist circumference, systolic and diastolic blood pressure, triglycerides, LDL cholesterol, HDL cholesterol, total cholesterol, C-reactive protein and interleukin 8.

**Results:**

There was a significant reduction in waist circumference (102.1±7.5cm to 100.8±7.4cm; p=0.03) and in body mass index (29.7±3.2kg/m^2^ *versus* 29.3±3.5kg/m^2^; p=0.03). Systolic blood pressure dropped from 141±18 to 129±13mmHg and diastolic from 79±12 to 71±10mmHg (with p<0.05 for both). No changes were observed on total cholesterol, LDL cholesterol and triglycerides, although HDL cholesterol levels improved, from 45.5±6.0 to 49.5±9.8mg/dL (p=0.02). There was a trend toward reduction of C-reactive protein (8.3%; p=0.07) and interleukin 8 levels (17.4%; p=0.058). The improvement in cardiovascular capacity was demonstrated by an increase of 13% in estimated volume of oxygen (p<0.001).

**Conclusion:**

Benefits of aerobic exercise of moderate intensity were seen within only 12 weeks of training in sedentary patients with metabolic syndrome. Considering the easy self-applicability and proven metabolic effects, an exercise program could be a first approach to sedentary patients with metabolic syndrome.

## RESUMO

**Objetivo:**

Avaliar se um programa de exercícios de intensidade moderada por um pequeno período pode alterar os parâmetros inflamatórios e melhorar os diferentes componentes da síndrome metabólica em pacientes sedentários.

**Métodos:**

Dezesseis pacientes completaram 12 semanas de exercício supervisionado, que consistiu em caminhar 40 a 50 minutos 3 vezes por semana, atingindo 50 a 60% da frequência cardíaca de reserva. Os parâmetros avaliados pré e pós-intervenção foram circunferência de cintura, pressão arterial sistólica e diastólica, triglicérides, LDL-colesterol, HDL-colesterol, colesterol total, proteína C-reativa e interleucina 8.

**Resultados:**

Houve redução significativa na circunferência da cintura (102,1±7,5cm *versus* 100,8±7,4cm; p=0,03) e índice de massa corporal (29,7±3,2kg/m^2^ *versus* 29,3±3,5kg/m^2^; p=0,03). A pressão arterial sistólica diminuiu de 141±18 para 129±13mmHg e a diastólica de 79±12 para 71±10mmHg (p<0,05 para ambos). Não foram observadas alterações no colesterol total, LDL-colesterol e triglicérides, mas houve melhora significativa nos níveis de HDL-colesterol, – 45,5±6,0 *versus* 49,5±9,8mg/dL (p=0,02). Houve uma tendência na redução de proteína C-reativa (8,3%; p=0,07) e interleucina 8 (17,4%; p=0,058). A melhora na capacidade cardiovascular foi demonstrada por um aumento de 13% no volume de oxigênio estimado (p<0,001).

**Conclusão:**

Os benefícios do exercício aeróbico com intensidade moderada puderam ser observados em apenas 12 semanas de treinamento em pacientes sedentários com síndrome metabólica. Considerando a facilidade de autoaplicabilidade e os efeitos metabólicos comprovados, tal programa pode ser uma primeira abordagem para pacientes sedentários com síndrome metabólica.

## INTRODUCTION

The metabolic syndrome (MS) is a complex disorder represented by a cluster of pro-atherogenic metabolic abnormalities, which are commonly associated with abdominal obesity and insulin resistance, such as dysglycemia, reduction of HDL cholesterol (HDL-c), increase of triglycerides, hypertension and presence of a pro-inflammatory state^(1,2)^. These factors predispose patients to a higher risk of developing atherosclerotic disease, with significant increase in morbimortality^(3,4)^. It is estimated that approximately 23% of Americans and 15% of Europeans have MS^(5)^. In Brazil, national data from a rural area shows an overall prevalence of 30% and adjusted prevalence by age based on the Brazilian population census of 25%^(6)^.

Benefits of regular exercise in global health are widely known. In men, the improvement of physical capacity in one metabolic equivalent is associated with a reduction of total mortality of 13%^(6)^. In addition to diet and weight loss, physical exercise should be the cornerstone in the management of MS^(7,8)^. Nonetheless, physical inactivity is a highly prevalent feature on overweight and obese individuals^(9)^. Regular physical activity can reduce systemic blood pressure^(10)^, the need for insulin, total body fat, adhesiveness and platelet aggregation, serum levels of triglycerides and increase levels of HDL-c^(11)^. Although these effects were already evaluated individually, only recently effects of exercise on MS have been described^(12,13)^.

Clinical guidelines often suggest that physical activity should be performed in a moderate intensity^(14)^, but no consensus exists about the ideal exercise prescription in this population. In the same way, we were unable to find in the literature a stepwise approach that could facilitate adherence to physical activity in this generally sedentary population^(15)^. In real clinical situations, outside of controlled environment of clinical studies, the easily to implement the program, allowing a long-term high-adherence associated with a rapid improvement in anthropometric parameters, should be an important point to be considered^(16)^.

## OBJECTIVE

This pilot study evaluated the short-term effects of a moderate intensity exercise program, performed three times a week, in cardiometabolic and inflammatory profile of sedentary patients with metabolic syndrome.

## METHODS

### Population

Sixteen patients with MS were recruited from an outpatient setting in a tertiary cardiac center. All patients signed the form of consent prior to the investigation. This study was approved by the Institutional Review Board of the *Pontifícia Universidade Católica do Paraná* under the number 1,274/06.

### Inclusion and exclusion criteria

The diagnosis of MS was established according to the parameters defined by the International Diabetes Federation^(17)^, where the presence of abdominal obesity is mandatory defined as a waist circumference ≥80cm for women and ≥94cm for men associated with two or more of the following factors: high blood pressure (systolic blood pressure - SBP≥130mmHg or diastolic blood pressure - DBP≥85mmHg), fasting plasma glucose ≥100mg/dL, triglycerides ≥150mg/dL and low HDL-c (<40mg/dL in men <50mg/dL in women). We included only sedentary patients, defined as those who performed physical exercise for less than 30 minutes and less than three times per week^(18)^, without restrictive or obstructive pulmonary disease, no osteoarticular and/or muscle limitation, preserved renal function (creatinine <1.5mg/dL) and body mass index (BMI) <40kg/m^2^. Patients recently enrolled in any other kind of physical activity program were excluded. Those who were unable to complete the proposed program of 12 weeks and who had myocardial ischemia during the initial treadmill tests were also excluded.

### Study protocol

To perform this pilot, prospective, pre-post study, we originally carried out a database analysis of patients from an ambulatory setting in a single tertiary cardiac care center. Potential candidates were contacted by telephone to explain the objectives of the study. For those who expressed interest, an initial assessment was scheduled in order to explain the study. After participants read and signed the informed consent, an initial clinical evaluation was performed, and clinical and anthropometric data were recorded. All patients had to have a negative functional test for myocardial ischemia from the last 12 months. Those who did not have this type of screening in the last 12 months were submitted to a treadmill test. Patients with abnormal test result were referred for appropriate clinical investigation and excluded from our study.

Participants underwent a blood sample collection (at least 10 hours of fasting), in the week before starting the first session of the physical activity program and no longer than 3 days after the last session, to evaluate the following inflammatory and metabolic parameters: blood glucose, triglycerides, total cholesterol, HDL-c, ultra-sensitive C-reactive protein (us-CRP) and interleukin 8 (IL-8). LDL cholesterol (LDL-c) was calculated using Friedwald formula^(19)^. All exams, excepted the IL-8, were performed in the same laboratory. Serum levels of IL-8 were measured by ELISA with commercially available antibodies (R&D Systems, Minneapolis, USA). The concentrations of the chemokine (pg/mL) were calculated with reference to standard curve performed with the corresponding recombinant molecule. The range of detection of this method varies from 31.25 to 2,000pg/mL. This analysis was performed by AMS in a university facility where the authors are affiliated.

For assessment of cardiopulmonary capacity we performed the 1-mile walk test (1,600m), which consists of walking 1,600m in less time possible without trotting or running^(20)^. Time to complete the walk and heart rate (HR) at the end of the 1,600m were recorded. To calculate the maximum volume of oxygen (VO_2max_), we applied the following formula:


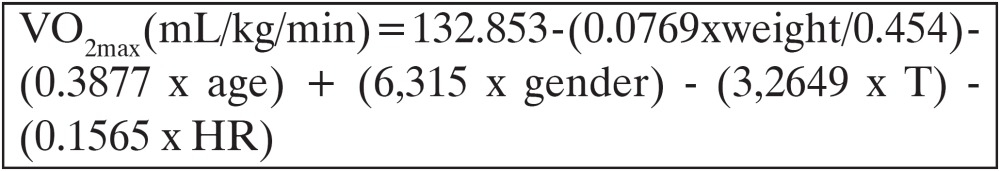


In this formula: weight indicates bodyweight, in kg; age was indicated in years; sex was indicated as 1, if male, and as 0, if female; T indicates time to complete the route in minutes and hundredths of minute; HR indicates HR at the end of 1,600m. The test was performed at the beginning and at the end of the program, at the same track where the program of physical activity was developed.

### Physical exercise program

The participants were submitted to a program of physical conditioning three times a week, in an outdoor athletic track in a public square, under the supervision of a physiotherapist and a fitness trainer. To enable better monitoring, the patients participated in groups of five or six people. The sessions had duration of 1 hour, being 5 minutes of warm-up, 40 to 50 minutes of cardiovascular stimulation (walking aiming to maintain the training HR – THR) and 5 minutes of cool down. During all session patients used HR monitors (Polar^®^) to assure that the target HR was reached according to individual calculations. Each participant had a badge with their name and values of the HR to be achieved. To calculate the THR, the following formula was applied:


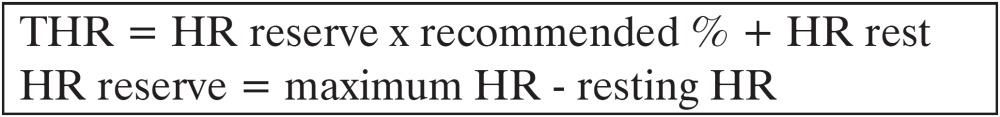


Maximum HR was determined as 220 – age and HR at rest was measured after 5 minutes of lying down. Physical activity was prescribed for this population using a HR reserve of 50 to 60%, that characterizes moderate intensity physical exercise. For patients who used beta-blockers, exercise prescribed was done based on table described in the I National Cardiovascular Rehabilitation Consensus^(21)^, which established a correlation between the dose of beta-blockers and lower percentage of HR. After 12 weeks of aerobic training (36 sessions) patients were assessed again to obtain clinical and metabolic data.

### Statistical analysis

The nature of categorical variables was expressed by frequency and percentage of cases. Variables of quantitative nature were expressed as means and standard deviations. For these variables, normal condition was evaluated by Shapiro-Wilk's test. We used the Student's t-test for paired samples to compare the parameters before and after 12 weeks of training. Values of p<0.05 indicated statistical significance.

## RESULTS

Of 24 patients initially enrolled, 2 who had positive exercise test for myocardial ischemia were excluded, and referred for appropriate clinical investigation. One patient had a muscle injury during sessions and was also excluded. Another five individuals left the study for personal reasons. Therefore, 16 patients completed the program.

Table 1 shows the baseline characteristics of population and medications used by the time of initial evaluation. All patients received care at the outpatient unit regardless of the physical activity program. Therefore, when necessary, changes in dosing regimens were performed by medical assistants. Only four patients had to change the therapeutic regimen: one patient suspended the medication by his own decision; one patient's beta-blocker was replaced by an inhibitor of angiotensin-converting enzyme; other received an increase of 5mg in the dose of amlodipine; and for another patient the simvastatin was suspended. There was no nutritional guidance during the study but patients previously followed by a nutritionist were encouraged to continue the treatment (n=4).

**Table 1:** Baseline characteristics of subjects (n=24)

Characteristics	Subjects n (%)
Gender (male)	5 (31.25)
Age (mean, years)	60±8.5
Hypertension	15 (93.75)
Diabetes	6 (37.5)
Previous history of cardiovascular disease	2 (12.5)
Smoking	4 (25)
Ex-smoking	2 (12.5)
Obesity (BMI>30kg/m^2^)	6 (37.5)
Overweight (BMI>25kg/m^2^)	9 (56.25)
Statin	10 (62.5)
Fibrates	3 (18.75)
Oral hipoglycemiant	5 (31.25)
Acetylsalicylic acid	4 (25)
Inhibitor of angiotensin-converting enzyme	9 (56.25)
Beta-blocker	7 (43.75)
Diuretic	5 (31.25)
Angiotensin II receptor antagonists	3 (18.75)
Amlodipine	3 (18.75)

BMI: body mass index.

In relation to the anthropometric data, we observed a slight weight reduction (-1.5%; p=0.034) and a decrease in abdominal circumference (-1.3%; p=0.03) and BMI (-1.5%; p=0.03). There was significant improvement in SBP (-7.0%; p=0.04) and in DBP (-9.3%; p = 0.005). We did not observe any changes in total cholesterol, LDL-c and triglycerides levels, however, a significant increase was seen in HDL-c from 45.5±6.0mg/dL to 49.5±9.8mg/dL (p=0.02). A small increase in glucose fasting mean was seen, but it was within the range of glucose intolerance. To inflammatory parameters, we observed a reduction of 8.3% in the levels of CRP (from 3.0±2.2 to 2.2±1.3; p=0.07) and of 17.4% in IL-8 (from 84.8±55.5 to 48.8±18.8; p=0.058), both showed a trend toward statistical significance. All these changes were associated with an improvement in cardiovascular capacity demonstrated by the increase in VO_2max_: 24.8±8.5mL/kg per minute to 28.8±9.8mL/kg per minute (p<0.001). Table 2 shows means, standard deviations and percentage change of variables before and after the physical exercise program.

**Table 2:** Effects of a 12-weeks program of physical exercise on clinical and metabolic parameters (data of 16 patients who completed the program; 5 men and 11 women)

Variable	Baseline	End of program	Mean of variations (%)	p value
Weight (kg)	79.1 (±9.8)	78.0 (±10.1)	-1.5	0.034
WC (cm)	102.1 (±7.5)	100.8 (±7.4)	-1.3	0.031
BMI (kg/m^2^)	29.7 (±3.2)	29.3 (±3.5)	-1.5	0.034
SBP (mmHg)	141.1 (±17.9)	128.6 (±12.9)	-7.0	0.047
DBP (mmHg)	79.1 (±12.0)	71 (±9.6)	-9.3	0.005
Fasting glucose (mg/dL)	105.4 (±39.2)	115.0 (±38.5)	10.3	0.0005
Total cholesterol (mg/dL)	204.8 (±41.8)	217.8 (±56.5)	6.2	0.136
HDL cholesterol (mg/dL)	45.5 (±6.0)	49.5 (±9.8)	8.4	0.024
LDL cholesterol (mg/dL)	126.7 (±38.2)	138.6 (±47.1)	9.6	0.087
Triglycerides (mg/dL)	162.8 (±89.4)	148.4 (±69.0)	-4.5	0.569
CRP-us (mg/dL)	3.0 (±2.2)	2.2 (±1.3)	- 8.3	0.077
IL-8 (pg/mL)	84.8 (±55.5)	48.8 (±18.8)	-17.4	0.058
VO_2max_ (mL/kg per min)	24.8 (±8.5)	28.8 (±9.8)	13.0	0.0001

WC: waist circumference; BMI: body mass index; SBP: systolic blood pressure; DBP: diastolic blood pressure; us-CRP-us: ultra-sensitive C-reactive protein; IL-8: interleukin 8; VO_2max_: maximum volume of oxygen.

## DISCUSSION

In this study we demonstrated that, even in short term, a reasonable program of exercise, performed three times a week, was able to significantly improve cardiometabolic profile of patients. Recent data show a significant reduction in cardiovascular mortality in the past decades in several countries^(22)^. However, this scenario could be better if not for a significant increase in the incidence of obesity and its complications, like diabetes^(23)^. Abdominal obesity constitutes an important risk factor for cardiovascular disease^(24)^, and it is often associated with the pro-atherogenic metabolic changes that characterize the MS. Similarly, sedentary behavior is also a common feature in MS patient, and to defeat it is a challenge for health professionals. A long-term change in lifestyle can halt the evolution of MS. Gayda et al.^(25)^ had demonstrated a great benefit in metabolic profile, even in patients with metabolic syndrome and established coronary artery disease (CAD) in a program combining physical exercise and nutritional guidance. In addition, in their study, 20% of patients without CAD no longer had diagnostic criteria for MS after 12 months; in less severe patients, these numbers were even better: 31%. The need to associate nutritional guidance and physical activity prescription should be emphasized especially for obese patients^(26)^.

The concomitant improvement of multiple risk factors of MS patients participating in many different exercise programs have been poorly investigated so far. The Studies of a Targeted Risk Reduction Intervention through Defined Exercise (STRRIDE) evaluated the effect of different strategies in exercise prescription on decreasing the prevalence of MS^(27)^. In that study, which included 334 participants, only 171 addressed all components of MS. These sedentary overweight or obese individuals had been randomly allocated into control (n=41) or program of low volume/moderate intensity (n=41, 19km per week, 40 to 55% of VO_2_ peak), low volume/high intensity (n=45, 19 km per week, 65 to 80% of VO_2_ peak) and high volume/high intensity (n=44, 32 km per week, 65 to 80% of VO_2_ peak). The benefits obtained after 8 to 9 months of training, including 2 to 3 months of initial progression of the exercise, were significant for the low volume/ moderate intensity program, and were even larger in the high volume/high intensity group^(28)^. Our study applied a moderate intensity exercise program, with an even smaller amount compared with STRRIDE study. In addition, our study duration was 3 months only. We noticed a favorable effect on weight, BMI, abdominal circumference, blood pressure and HDL-c. The moderate intensity exercise of the STRRIDE study showed a significant effect only in abdominal circumference and serum levels of triglycerides, while the effects on BMI, HDL-c and blood pressure were only found in the group of high intensity/high volume. These differences may be explained by the fact that in STRRIDE only 40% of enrolled patients had MS according to ATP III criteria. It is possible that a less favorable metabolic profile in the initial patients in our study had contributed to these changes that took place at an early stage.

The benefits found in our study concerning reduction of SBP and DBP levels were quite large considering the short period of intervention and the no specific dietary guidance. A previous study that combined diet and exercise and then compared results with diet alone, showed a synergism when both, diet and exercise, are incorporated in lifestyle. The exercise was prescribed for this population using 60 a 80% of HR reserve with duration of 40 minutes, 2 times per week^(10)^.

One of the main characteristics of MS is the increase of pro-inflammatory activity, clinically evidenced by increased serum inflammatory markers, such as CRP and IL-8. This increase is directly related to the number of components of MS^(29)^. Multiple mechanisms are correlated with the elevation of CRP and IL-8, and, in this case, there is a significant participation of obesity, because the excess of adipose tissue releases pro-inflammatory cytokines, that induce the production of these molecules^(30)^. This production, by the adipocyte or cells of the immune system outside the adipose tissue, has a central role in chronic inflammatory status in overweight people.

Few studies have evaluated the role of physical exercise in reduction of inflammatory cytokines, especially IL-8, in patients with MS. Trøseid et al.^(30)^ studied 32 sedentary and obese individuals with MS divided into 4 groups: the control group (n=6), the pravastatin 40mg/day group (n=8), the pravastatin associated with exercise group (n=9), and exercise only group (n=9). The program of physical exercise was performed 3 times per week with session of 45 to 60 minutes each. In addition, aerobic exercises and exercises for muscle strength were done in proportion of 40% and 60% of time, respectively. After 3 months, there was a reduction of IL-8 in groups that exercised when compared with control group, and with the group that used only pravastatin. These anti-inflammatory effects of physical activity had already been described in other groups, including healthy seniors. Apparently, neither the age nor the health status are able to limit the ability of physical activity to reduce inflammation^(31)^. Our study also found this anti-inflammatory effect with a trend for reduction of 8.3% in the CRP levels and 17.4% of IL-8 levels.

An interesting finding was the increased levels of fasting plasma glucose in our patients. Other studies also do not mention improvements in glucose levels^(10,25,28)^. In STRRIDE study, no significant difference in fasting blood glucose levels pre-and post-training was reported, but there was a significant increase in the index of insulin sensitivity, suggesting that the first is not a sensitive parameter of improved insulin sensitivity. A possible explanation for high fasting plasma glucose level and the absence of low triglycerides levels in our study might be for the inadequate intake of carbohydrates during evaluation, mainly because we did not have any diet control.

Our study had three limitations that merit consideration. First, because all patients were continuously seen by their assistant physicians, drug treatment changes were allowed at their discretion. Although, it is unlikely that the few changes made had caused any impact on results. Second, instead of ergoespirometric tests to calculate the VO_2max_, we estimated it performing the 1-mile walk test, which was proposed and validated for a normal population^(20)^. Moreover to underwent the test, patients should not use medicines that may interfere with HR behavior^(32)^. Despite this limitation, we used this tool in a comparative way to measure the comprehensive training effect. In this case, the test was applied to evaluate the improvement of cardiorespiratory conditioning. Even if the test applied was questionable, we could also use duration of the test as a measure of improvement. All patients reduced significantly the time they took to run the 1.600m after the training program (data not shown).

Finally, the third limitation in this study may be the lack of a control group. However, we have considered unethical to enroll patients and advise them not to perform physical activity. This strategy of not including a control group is justified in studies involving high risk patients^(33)^. In the same way, as the aim of this study was to evaluate a program that could be a first approach for sedentary patients, its comparison with more intensive programs would not be useful. Although the duration of our program was certainly shorter than currently indicated for long-term cardiovascular prevention, the program was efficient to improve cardiometabolic profile of sedentary patients with MS in a short period of time. Therefore, it may be a useful initial tool for exercise prescription.

## CONCLUSION

Our results showed that performance of moderate intensity aerobic exercise, with a program of easy feasibility, applied in a public square three times a week produced significant improvement in anthropometric, hemodynamic and metabolic parameters of sedentary individuals even in program of short period of time. This simple recommendation can be done by general practitioners and does not need specialized supervision, what is consistent with the currently recommendations that strategies of low cost and high impact should be implemented to control cardiovascular risk factors.
